# The Nerve Growth Factor Signaling and Its Potential as Therapeutic Target for Glaucoma

**DOI:** 10.1155/2014/759473

**Published:** 2014-08-31

**Authors:** Haitao Wang, Rikang Wang, Thilini Thrimawithana, Peter J. Little, Jiangping Xu, Zhong-Ping Feng, Wenhua Zheng

**Affiliations:** ^1^State Key Laboratory of Ophthalmology, Zhongshan Ophthalmic Center, Sun Yat-sen University, Guangzhou, Guangdong 510006, China; ^2^Discipline of Pharmacy, School of Medical Sciences and Diabetes Complications Group, Health Innovations Research Institute, RMIT University, Bundoora, VIC 3083, Australia; ^3^School of Pharmaceutical Sciences, Southern Medical University, Guangzhou 510515, China; ^4^Department of Physiology, Faculty of Medicine, University of Toronto, 1 King's College Circle, Toronto, ON, Canada M5S 1A8

## Abstract

Neuroprotective therapies which focus on factors leading to retinal ganglion cells (RGCs) degeneration have been drawing more and more attention. The beneficial effects of nerve growth factor (NGF) on the glaucoma have been recently suggested, but its effects on eye tissue are complex and controversial in various studies. Recent clinical trials of systemically and topically administrated NGF demonstrate that NGF is effective in treating several ocular diseases, including glaucoma. NGF has two receptors named high affinity NGF tyrosine kinase receptor TrkA and low affinity receptor p75NTR. Both receptors exist in cells in retina like RGC (expressing TrkA) and glia cells (expressing p75NTR). NGF functions by binding to TrkA or p75NTR alone or both together. The binding of NGF to TrkA alone in RGC promotes RGC's survival and proliferation through activation of TrkA and several prosurvival pathways. In contrast, the binding of NGF to p75NTR leads to apoptosis although it also promotes survival in some cases. Binding of NGF to both TrkA and p75NTR at the same time leads to survival in which p75NTR functions as a TrkA helping receptor. This review discusses the current understanding of the NGF signaling in retina and the therapeutic implications in the treatment of glaucoma.

## 1. Introduction

Glaucoma is one of the leading causes of blindness worldwide. Glaucoma is characterized by retinal ganglion cell (RGC) degeneration and loss of visual field and it occurs with or without elevated intraocular pressure (IOP) [1]. Apoptosis or programmed cell death of RGCs and optic nerve degeneration may be the cause of blindness and it may occur in the state of elevated intraocular pressure; however, both RGC apoptosis and optic nerve atrophy, due to glaucoma, can occur independently of elevated IOP. Clinically, in addition to the lowering of IOP, one of the main targets of glaucoma therapy is to delay the apoptosis and promote the survival of RGC. Up to now, there is considerable evidence showing that attenuation of RGC degeneration is potentially an effective therapeutic strategy for treatment of glaucoma [2, 3]. Thus, therapeutic neuroprotection of RGCs aims to prevent or delay cell death and maintaining normal neuronal functions is an important alternative approach for the treatment of glaucoma.

Nerve growth factor (NGF) was discovered in 1948. It prevents neuronal apoptosis in primary cultured neurons and reduces neuronal degeneration in animal models of neurodegenerative diseases [4]. These results in animals have led to several clinical trials [5, 6]. In clinical studies, treatment with NGF was accompanied by beneficial effects on cognitive performance, but it also led to back pain [7]. Positive results from the use of NGF in the treatment of classical neurodegenerative diseases lead researchers to investigate the role of NGF of the treatment of glaucoma based on glaucoma being a neurodegenerative disease related to the damage of optic nerves. RGCs are special neurons which receive visual information from photoreceptors and transmit signals to several brain regions including the thalamus, hypothalamus, and mesencephalon and midbrain [8]. Although NGF treatment is effective in the treatment of glaucoma, in some studies, there are also some negative reports; one example is the proapoptotic effect of p75 neurotrophin receptor (p75NTR) in glia cells; binding of NGF to p75NTR is associated with retinal ganglion cell apoptosis [9]. Thus, an enhanced understanding of the molecular pathways and mechanisms is required to better appreciate and potentially exploit the therapeutic potential NGF and its signaling pathways for the treatment of glaucoma. In this review, we will examine the current understanding of the NGF signaling pathway and its potential as a therapeutic target for the treatment of glaucoma.

## 2. Nerve Growth Factor Receptors and Their Signaling Pathways

### 2.1. General Features of NGF and Its Similarity with Other Growth Factors

Growth factors are produced by our body and they have a very extensive role in the regulation of many cellular processes. The binding of growth factors to their receptors on the cell surface affects cellular survival, proliferation, and/or differentiation [10, 11]. For example, platelet-derived growth factor promotes the proliferation of glioblastoma cells through downregulation of miR-21 [12]; the breadth of actions of such an agent is apparent from the observation that it can also enhance glycosaminoglycan elongation on the proteoglycan biglycan and thereby plays a role in the initiation of atherosclerosis [13]. The pleiotropic growth factor, transforming growth factor-*β*, promotes wound healing, inhibits macrophage proliferation, and protects against nerve-injury-induced neuropathic pain [14].

NGF is another important polypeptide growth factor which functions to regulate the growth and survival of nerve cells; it was discovered by Rita Levi-Montalcini and Stanley Cohen in the 1950s [15]. NGF belongs to a family of factors also known as neurotrophins. Other members of the neurotrophin family include brain-derived neurotrophic factor (BDNF), neurotrophin-3 (NT-3), and neurotrophin 4/5 (NT-4/5); all of them are known for regulating brain development and functions [16]. NGF is formed by cleavage from Pro-NGF, which is the precursor protein form of NGF; however, the roles of Pro-NGF and NGF are not consistent; treatment with Pro-NGF in cervical ganglia neurons which express both of the NGF receptors p75NTR and tyrosine receptor kinase A (TrkA) leads to programmed cell death, whereas NGF treatment of these same neurons results in survival and axonal growth [17]. Free NGF displays multiple physiological actions, in the central nerve system. NGF possesses neurotrophic effects and is critical for the neurite outgrowth and survival and maintenance of neurons. Studies,* in vitro* and* in vivo*, have shown that NGF stimulates neurite outgrowth and axonal branching and extension [18–20]. Most importantly, NGF has strong antiapoptotic effect and, with deprivation of NGF, neurons exhibit a series of morphological changes and eventually undergo apoptosis [21].

The clinical significance of NGF has been widely studied and it is recognized that NGF profoundly affects the development of both the young and the adult nervous systems [22]. In the central nervous system, NGF is a key neurotrophin and its dysregulation could be involved in various neuronal degeneration diseases such as Alzheimer's disease and multiple sclerosis [23, 24]. Dysfunction of NGF may also be linked to mental or psychiatric disorders, such as schizophrenia, depression, and autism [25–27]. Low levels of NGF in cerebrospinal fluid and a deficit of NGF signaling might provide the basis for the occurrence of these neurological diseases [27]. Besides its role in the CNS, there is evidence that NGF circulates throughout the body and plays roles in many organs [28]. For example, variability in NGF levels is associated with atherosclerosis and hence cardiovascular disease and also metabolic disorders such as diabetes and obesity [29, 30]. Specifically, NGF levels are decreased in atherosclerotic coronary vascular tissue and a decrease in plasma NGF could be detected in metabolic syndrome patients [31]. NGF deficits are the main cause of these diseases mentioned above, so, to some extent, supply of NGF to the target region may reverse the pathology of the diseases or alleviate the symptoms. However, the obstacle is the drug delivery technology and pharmacokinetic properties since the apparent need is to deliver NGF to the target region and specifically the target regions in order to reduce the adverse effects at other sites [32]. This obstacle will not occur if NGF is used topically, for example, in the application of NGF in the treatment of ocular disease, and we will illustrate these possibilities in the following sections.

NGF plays its role by binding to its receptors located in the surface of cells. TrkA is the high affinity catalytically active receptor for NGF. NGF binding to TrkA leads to the phosphorylation of TrkA and activation of its downstream targets, such as protein kinase B (Akt) or extracellular signal-regulated protein kinase 1/2 (ERK1/2), which eventually cause neural differentiation and prevention of apoptosis [33]. The other NGF receptor, p75NTR, is a low affinity receptor [34]. The precise role of p75NTR is complicated, and, depending upon the cellular context, it can promote cell survival, cell death, or growth inhibition; for example, treatment of normal eyes with an NGF mutant-selective p75NTR agonist causes progressive RGC death [35]; in contrast, p75NTR overexpression in breast tumor cells favors tumor survival and contributes to tumor resistance to drugs [36].

### 2.2. Signaling Pathway of NGF and Its Receptors

**Figure 1:**
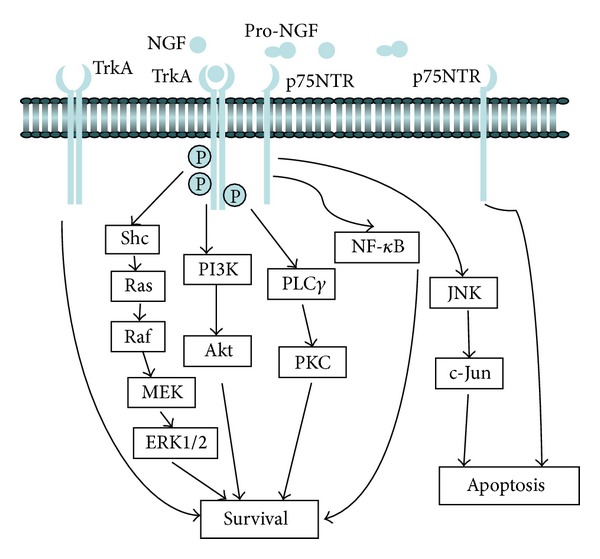
Signaling of NGF receptors. NGF is formed by cleavage from Pro-NGF, which is the precursor protein form of NGF. TrkA receptor is the high affinity receptor for NGF; NGF binding to TrkA causes the phosphorylation of TrkA and activation of multiple signaling pathways such as the PI3K/Akt, Ras/Raf/MEK/ERK1/2, or PLC*γ*/PKC signaling pathways. Activation of these pathways eventually leads to different biological functions including the prevention of apoptosis. The other NGF receptor, p75NTR, is a low affinity receptor. The precise role of p75NTR depends upon the cellular context; it can enhance cell survival through NF-*κ*B pathway or promote cell death through JNK/c-Jun signal pathway.

The affinity of NGF binding to p75NTR receptor is weaker than NGF binding to TrkA, but the cell type distribution of p75NTR is wider than that of TrkA; TrkA receptor is mainly expressed at neurons responsive to NGF: peripheral sensory neurons, sympathetic neurons, and basal forebrain cholinergic neurons [37], while the p75NTR receptor is more widely distributed. In addition to cells expressing the TrkA receptor, the p75NTR receptor could be detected in motor neurons, Schwann cells, and cerebellar Purkinje cells [38]. Signaling of NGF receptors is shown in Figure 1.

TrkA is the high affinity catalytic receptor for the NGF and it mediates the main effects of NGF, which include cell growth, the formation and regeneration of neurites, and avoidance of programmed cell death [39]. Binding of NGF to TrkA receptor facilitates receptor dimerization and autophosphorylation of its tyrosine residues. Activation (phosphorylation) of the TrkA receptor provides docking sites for effector molecules such as Shc which in turn induces the recruitment of a complex of Shc/Grb2, subsequent to which several downstream signaling cascades are initiated and propagated [40].

Phosphorylation of the TrkA receptor leads to the interaction of TrkA and phosphatidylinositiol-3 kinase (PI3K). PI3K is activated and recruited to the plasma membrane and leads to the production of phosphoinositide 3,4,5-trisphosphate and membrane translocation of the serine/threonine-protein kinases Akt and Akt activation [20]. Although there are other downstream targets of PI3K, the PI3K/Akt signaling pathway is particularly important for neuronal survival and the synthesis of many new cellular proteins. In one exemplar, pathway Akt phosphorylates the proapoptotic proteins, such as forkhead box-O transcription factors (FoxO) and B-cell lymphoma 2 family members, thereby inhibiting neuronal apoptosis [41–43]. FoxO is a classic target of growth factor Trk receptor signaling [44, 45]; FoxO proteins are expressed in the detached retina [46]; therefore, phosphorylation of FoxO mediated by Akt may play a role in neurotrophins-mediated cell survival.

Another NGF-activated signaling pathway is the Ras-mediated activation of the mitogen-activated protein kinase (MAPK) pathway, which is initiated through recruitment and phosphorylation of Shc [47]. Ras is a membrane-associated G protein; the active Ras protein binds to and phosphorylates several proteins, including the protooncogene Raf. Raf, in turn, activates MAPK kinase (MEK) and phosphorylated MEK activates ERK1/2 [48]. Phosphorylated ERK1/2 may enter into nucleus and regulate the activity of many transcription factors including ETS domain-containing protein ELK1 [49]. ERK1/2 may also phosphorylate ribosomal S6 kinase (S6K), which leads to the phosphorylation of cyclic adenosine monophosphate response element binding protein, eventually affecting the regulation of the expression of NGF-inducible genes and, thus, contributing to neuronal differentiation or neurite outgrowth [50]. Besides the two pathways mentioned above, TrkA activation also leads to the survival and growth of neuronal cells through Phospholipase C gamma1 (PLC*γ*1) [51]. PLC*γ*1 supports activation of PKC signaling pathway and is thus involved in antimitogenic/mitogenic signaling.

The other receptor for NGF is p75NTR, but it is not a specific receptor for NGF as it also binds other neurotrophins, such as NT-3, NT-4/5, and BDNF [52]. The role of the p75NTR receptor is complicated. Binding of NGF with p75NTR with low affinity leads to apoptosis or cell survival in different cellular contexts [35, 36]. p75NTR can induce apoptosis both* in vitro* and* in vivo*. p75NTR activates Rac GTPase and activated c-jun N-terminal kinase (JNK), including an injury-specific isoform, JNK3 [53]. JNKs stimulate the expression of proapoptotic genes via the transactivation of specific transcription factors [54], so p75NTR can promote cell death. However, it also enhances cell survival; NGF treatment activates nuclear factor *κ*B (NF-*κ*B) through p75NTR and during this process, p75NTR-mediated NF-*κ*B activation enhances the survival response of developing sensory neurons to nerve growth factor [55]. NF-*κ*B is a nuclear transcription factor that regulates expression of a large number of genes that are critical for the regulation of cell survival. Thus, in summary, the p75NTR receptor activates apoptotic signaling through the JNK cascade or cell survival through NF-*κ*B pathway.

NGF binds to both TrkA and p75NTR receptors when they coexpressed on the outer cell membrane, even though the affinity of TrkA with NGF is much higher. Studies* in vitro* have shown that neurons coexpressing p75NTR and TrkA respond to lower concentrations of NGF [56, 57], which means that p75NTR increases the responsiveness of TrkA to NGF. When the two receptors are coexpressed, the rate of association of NGF with TrkA increases compared to cells expressing TrkA alone [58]. The result infers that this interaction leads to the formation of binding sites with higher affinity for NGF than that of either receptor alone. Structural and mechanistic insights into NGF interactions with the TrkA and p75NTR receptors indicate that NGF could dimerize TrkA and p75NTR exists as a preformed oligomer that is not dissociated by NGF [59]. There is no evidence to show that TrkA and p75NTR interact directly, so they may interact indirectly through the convergence of downstream signaling pathways and/or share adaptor molecules, rather than through direct receptor-receptor interactions [59]. These data indicate that the final fate of cells coexpressing both TrkA and p75NTR is complicated with the functional response related to the abundance of each receptor and the different agonists.

## 3. Causes and Pathophysiology of Glaucoma

Glaucoma is a severe eye disease, which is usually associated with absolute or relative elevated fluid pressure within the eyes and gradually progressive visual field loss. One major risk factor for glaucoma is the raised IOP. However, other mechanisms must be involved in the pathology of glaucoma because glaucoma can develop in the absence of elevated IOP and, in the clinic, different individuals respond differently to the elevated pressure [60]. In some populations, patients may have high eye pressure for many years but this never leads to glaucoma. Molecular studies on the differential expression of human genes under conditions of elevated IOP revealed that MMP1, MMP10, CXCL2, and PDPN were general responders and were altered in almost all the patients with glaucoma whilst STATH, FBN2, TF, OGN, IL6, IGF1, CRYAB, and ELAM1 (marker for glaucoma) had very patient specific changes in expression [61].

**Figure 2:**
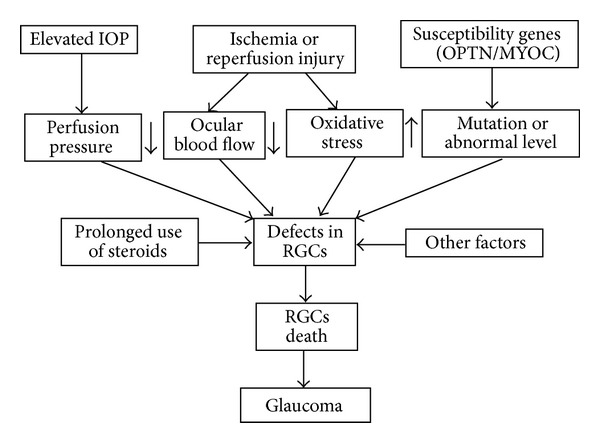
A model of insults and their involvement of glaucoma. Increased intraocular pressure (IOP) usually leads to abnormal pressure-flow relationship; periods of ischemia are then more likely to occur when ocular perfusion pressure is reduced leading to increased oxidative stress due to the reactive oxygen species. These insults lead to the impairment of RGCs and eventually lead to RGC's death and glaucoma. Functional defects caused by mutations in susceptibility genes, such as OPTN/MYOC, could also lead to defects in RGCs and contribute to the pathogenesis of glaucoma. Other factors, such as topical ocular administration of steroids, are the most likely to cause alteration of IOP and increase the risk of developing glaucoma.

Notwithstanding the above, among the several causes for glaucoma, elevated eye pressure is one of the most important and best recognized acceptable risk factors [62]. Besides elevated IOP, glaucoma is also thought to arise from a mutation in a single gene or a group of genes; for instance, it is well identified that MYOC mutations are a major cause of glaucoma [63, 64]. This notion is supported by the observation that people with a family history of glaucoma have higher risk of developing glaucoma. The relationship between glaucoma with gene mutations and phenotypes has been reviewed by Fan and Wiggs [65]. Other risk factors for glaucoma include severely restricted blood flow to the eye, prolonged use of steroids, and ethnic and gender factors.  Figure 2 shows a model of insults and their involvement of glaucoma.

Optic nerve damage is a common optic neuropathy of glaucoma, which is characterized by loss of retinal ganglion cells [66]. Both the high pressure and relatively low pressure can develop nerve damage and lead to blindness if left untreated. The inconsistent relationship of glaucomatous optic neuropathy with ocular hypertension has provoked the generation of other hypotheses and investigations. Among these studies, excitatory neurotransmitter toxicity (such as excessive glutamate release), hypoperfusion, trophic factors, retinal ganglion cell/axon degeneration, and neuron loss have received attention [67–70]. Neuroprotection is an effective strategy to attenuate RGC degeneration and facilitate the survival of optic nerves through blocking these risk factors associated with RGC loss in glaucoma. NGF plays an effect by preventing neuronal degeneration in animal models of neurodegenerative diseases [71, 72], so the application of NGF as a neuroprotective strategy for the medical treatment of glaucoma should be reasonable.

## 4. Interaction between NGF and Glaucoma

### 4.1. Glaucoma Alters the Expression of NGF and Its Receptors in Retinal Ganglion Cells and Visual Cortex

In normal conditions, primary cultured RGCs and transformed RGC-5 cells express various neurotrophins, such as brain-derived neurotrophic factor (BDNF), NGF, neurotrophin-3 (NT3), neurotrophin-4 (NT4), and also their relevant receptors, such as TrkA, p75NTR, or TrkB. RGC-5 cells also secrete NT3, BDNF, NGF, and NT4 into the cultured media [73]. In the visual system, the secretion of neurotrophins and the expression of their receptors maintain the morphological development; for example, neurotrophins and their receptors have been shown to exert various influences on guiding the morphological differentiation of neurons and controlling the functional plasticity of visual circuits; they may also participate in visual connectivity and the transmission of images into brain through optic nerve fibers [74].

While, in pathological conditions, such as glaucoma, the increased IOP will alter the level of NGF and NGF receptor expression, glaucoma significantly reduces the content of NGF in the cerebrospinal fluid (CSF) and lateral geniculate nucleus (LGN), but serum NGF protein levels may not be affected; this finding suggests that the NGF present in the CSF is most likely taken up by damaged retinal or brain neurons. Ongoing research shows that glaucoma increases the basal level of TrkA in the LGN and NGF administration further enhances this increase. However glaucoma had no effect on the expression of p75NTR at early stage, while NGF still enhanced the level of p75NTR; these results support the contention that glaucoma altered the basal level of NGF and NGF receptors in brain visual centers [75]. A relatively long term study showed that, after 7 days of ocular hypertension, the content of retinal NGF increased but it did so transiently. However, the TrkA receptor is upregulated and expression is sustained for a long period in RGCs [76]. After 28 days of ocular hypertension, the level of retinal BDNF increased significantly, but the hypertension had no effect on its receptor TrkB [76]. The TrkC receptor was also enhanced in Müller cells but not in retinal ganglion cells even though the level of NT-3 remained unchanged. Expression of retinal p75NTR increased late at day 28 [76]. These results are consistent with the observation in the neurodegeneration; transgenic mice of neurodegeneration showed a lower level of NGF and NGF deficits elicit a progressive neurodegeneration [77, 78]. In Alzheimer's disease, NGF has been shown to be effective in preventing the onset of the central cholinergic deficit, so the role of NGF in RGC degeneration caused by glaucoma is certainly worthy of further investigation.

### 4.2. NGF Affects Synaptic Plasticity and Glaucoma-Associated Proteins in the Optic Nerve System

In the CNS, NGF possesses obvious effects on modulating neuronal inputs and thereby synaptic plasticity. NGF augmentation significantly enhanced cholinergic neuronal markers and facilitated induction of hippocampal long-term potentiation (LTP); moreover, blockade of endogenous NGF significantly reduced hippocampal LTP and impaired retention of spatial memory [79]. These findings provide evidence that NGF is essential for NGF hippocampal plasticity and learning. NGF also plays a role in neuronal plasticity in the optical system or visual cortex where monocular deprivation leads to decreased cell size and impairment of synaptic plasticity in visual cortex, while intraventricular administration of NGF prevents the cell shrinkage and demonstrated a substantial recovery of functional binocular connections [80]. This information indicates that neurotrophic factors may contribute to the regulation of experience-dependent modifications of synaptic connectivity in the visual cortex. Intracortical infusion of NGF into adult cat visual cortex can recreate ocular dominance plasticity, suggesting that NGF is also involved in the activity-dependent modification of synaptic connectivity in the adult brain [81]. GAP-43 and synaptophysin are two presynaptic elements in the visual cortex, which are highly related to synaptic plasticity. NGF treatment stimulates phosphorylation of GAP-43 and increases the level of synaptophysin immunoreactivity in adult visual cortex [82]. NGF treatment of the adult visual cortex modulates presynaptic terminals, possibly by inducing axonal sprouting and formation of new synapses, and these changes may play a role in the NGF-induced functional plasticity.

Optineurin (OPTN) and myocilin (MYOC) are two genes linked to glaucoma [83]. Rezaie et al. identified that OPTN may be an adult-onset glaucoma gene and speculated that wild-type optineurin played a neuroprotective role in the eye and optic nerve through the TNF-α pathway; accordingly, when OPTN was defective, it may lead to visual loss and optic neuropathy as typically seen in normal and high-pressure glaucoma [84]. This speculation was supported by the results that NGF treatment enhanced the endogenous levels of both OPTN and MYOC genes in PC12 cells [85]. These results demonstrate that NGF affects the expression of glaucoma-associated genes and their protein products. The data also indicate that NGF treatment may be an effective way to intervene in the process of glaucoma. However, the roles of OPTN and MYOC are complicated. OPTN overexpression induces an upregulation of the endogenous MYOC in both RGC and PC12 cells, while overexpressing MYOC does not affect the OPTN level. MYOC and OPTN contribute to the development of neurodegenerative glaucoma through different mechanisms. Overexpression of MYOC inhibits NGF-induced neurite outgrowth in both PC12 cells and RGC-5 cells, while transfection of optineurin leads to increased apoptosis [86]. The acute role of NGF and the glaucoma-associated proteins still needs further investigation and a specific gene knock-out mouse may serve this purpose. So far, a conditional knockout mouse line of OPTN has been generated and is available at the Wellcome Trust Sanger Institute [87]; Myoc-null mice have also been produced by Kim and coworkers [88]; these genetically altered mice are useful tools for understanding the physiological function of glaucoma-associated proteins; they can also be used to model the phenotype of glaucoma. In addition, these mouse lines provide a foundation for future efforts aimed at deciphering the role of NGF in the treatment of glaucoma.

### 4.3. Differential Effects Mediated by NGF Receptors in Glaucoma

As discussed above, the role of the receptors, TrkA and p75NTR, is not consistent in the CNS. It is widely accepted that sympathetic neuron survival is regulated positively by TrkA and negatively by p75NTR. The apoptotic effect of p75NTR signaling occurs only under suboptimal survival conditions, while TrkA-mediated responses occur when survival conditions are optimal [89–91]. In fact, the survival response to NGF is mediated by competitive signaling between TrkA and p75NTR; as mentioned previously, p75NTR induces NF-*κ*B and JNK activation, whereas TrkA mediates MAPK and Akt activation and when the two receptors are expressed together, TrkA blocks the p75NTR-mediated JNK activation, but NF-*κ*B is unaffected [88, 90].

In the optical system, the two receptors may also function differently. In normal adult retinas, TrkA is expressed in RGCs, whereas P75 is expressed in glia. In contrast to results from* in vitro* studies,* in vivo* studies indicate that NGF binds to TrkA and p75NTR but fails to promote the survival of axotomized RGCs in the adult retina; however, TrkA agonists showed robust neuroprotective effects [92]. Pharmacological inhibition of p75NTR or in p75NTR knockout mice showed enhanced survival of axotomized RGCs. A combination of NGF or TrkA agonists with p75NTR antagonists further potentiated RGC neuroprotection* in vivo* [93]. Treatment with a mutant NGF that only activates TrkA affords significant neuroprotection. Supporting results were obtained for a biological response modifier that prevents endogenous NGF and pro-NGF from binding to p75NTR. Treatment of normal eyes with an NGF mutant-selective p75NTR agonist causes progressive RGC death, and, in injured eyes, it accelerates RGC death [35]. In a model of experimental glaucoma, the loss of RGC is accompanied by increased retinal p75 and Bax expression [9]. In summary, much data support the idea that NGF can be neuroprotective when acting on neuronal TrkA receptors on RGCs but engagement of p75NTR on glial cells antagonizes this effect. However, p75NTR cannot be viewed simply as a proapoptotic factor as in some situations; p75NTR may be a neuroprotective molecule [94] and p75NTR has beneficial effects on myelin formation and regeneration [95]. Thus, it is apparent that the role of p75NTR is complex and we need to unravel the precise role of p75NTR under different conditions and its related molecules during retinal development and degeneration.

## 5. Application of NGF in Retina Neurodegeneration and Glaucoma

NGF is a widely studied growth factor which possesses significant neuroprotective effects on neurons against various insults. In the central nervous system, NGF deficiency is involved in age-related neurodegenerative diseases; a deficit in the signaling and/or transport of NGF also leads to neurodegeneration [96]. Treatment with NGF can attenuate cholinergic deficit and improve cognitive behavior in animals. Clinical studies with chronic NGF administration in patients with Alzheimer's disease normalized electroencephalograph patterns and improved performance in words recognition tests [97]. These researchers proposed that NGF or compounds that induce the expression of endogenous NGF may be useful in the treatment of Alzheimer's disease or other neurodegenerative diseases. The application of NGF in the treatment of neurodegeneration prompts us to explore whether NGF will produce similar role in the treatment of optic nerve degeneration.

Sustained elevated IOP causes RGC degeneration and results in cell death. In animal models, glaucoma is induced by the injection of hypertonic saline into the episcleral vein of the eyes. A rat model of glaucoma showed that glaucoma led to progressive degeneration of RGCs, with the loss of nearly 40% of these cells after 7 weeks of treatment. The RGC loss is associated with the downregulation of NGF and NGF receptor expression in the retina and ocular treatment with NGF significantly reduced the deficit induced by glaucoma [98]. Another study showed that using NGF as an eye drop can attenuate the optic nerve damage that accompanies glaucoma; these investigators induced glaucoma in rats and measured the survival of RGCs with and without NGF eye drops administered four times daily for 7 weeks; it was found that significantly more RGCs survived in the treated group. In three patients with advanced glaucoma, treatment with topical NGF produced an improvement in visual acuity, contrast sensitivity, and electrophysiological functions [99]. What we should keep in mind is that most of the growth factors, including NGF, have low bioactive stability in the body owing to their short half-lives and slow diffusion, thus limiting its use as a neuroprotective drug [100];* ex vivo* gene delivery or biologically stable small molecules that could bind and activate the TrkA signaling pathway are alternative strategies [101, 102]. From the angle of tissue engineering, two distinct strategies could be used for delivering growth factors: growth factors can be chemically bound into or onto the matrix; growth factors may infiltrate the material and make them available to cells. On the other hand, growth factors could also be physically encapsulated in the delivery system, and they can be released from synthetic extracellular matrix to target specific cell populations [103]; however, none has been evaluated for ocular applications. The most apparent advantage of NGF for the application in the clinic is its ability to penetrate to the retina when administrated topically. The application of NGF may open therapeutic perspectives for glaucoma and other neurodegenerative diseases.

## 6. Conclusions and Future Research

The ultimate goal of glaucoma research is to find new compounds that will not only normalize IOP but also arrest or even reverse apoptotic damage to the optic nerve and RGCs and slow the rate of progression of the disease. This review discussed current knowledge of glaucoma, with an emphasis on the NGF and NGF receptor(s) signaling pathways, and further explored the possibility of targeting the NGF signaling pathway as a strategy for the treatment of glaucoma. NGF offers the promise of actually restoring visual function through acting on the TrkA receptor; however, we should be cautious regarding the future of NGF-dependent treatments in the armamentarium of glaucoma therapy as most of the present studies were in animal models and none has reached clinical success. After more research and deeper mechanistic understanding a randomized, controlled glaucoma, clinical trials need to be performed to evaluate the therapeutic effect of NGF in the treatment of glaucoma and this also will involve an evaluation of the magnitude and occurrence rate of adverse effects. In the future, developments of novel pharmacological interventions for glaucoma may include the development of small molecules that are specific for TrkA receptors in the optical nerves. With protein chemistry entering into a new phase of “peptide design,” it is hoped that careful design of these small molecules may be able to limit biological activity to only the wanted effects and minimize the adverse effects. Other strategies such as chemical immobilization of NGF into or onto the matrix and physical encapsulation of NGF in the delivery system may also be promising in the future studies. Such novel ideas for trophic support are still developing and may provide future “smart drugs” for glaucoma.
